# Agreement Technologies for Energy Optimization at Home

**DOI:** 10.3390/s18051633

**Published:** 2018-05-19

**Authors:** Alfonso González-Briones, Pablo Chamoso, Fernando De La Prieta, Yves Demazeau, Juan M. Corchado

**Affiliations:** 1 BISITE Digital Innovation Hub, University of Salamanca, Edificio Multiusos I+D+i, 37007 Salamanca, Spain; fer@usal.es (F.D.L.P.); corchado@usal.es (J.M.C.); 2 Centre National de le Recherche Scientifique - Laboratoire d’Informatique de Grenoble (CNRS-LIG), University of Grenoble-Alps, 38000 Grenoble, France; yves.demazeau@imag.fr; 3 Department of Electronics, Information and Communication, Faculty of Engineering, Osaka Institute of Technology, 535-8585 Osaka, Japan; 4 Pusat Komputeran dan Informatik, Universiti Malaysia Kelantan, Karung Berkunci 36, Pengkaan Chepa, 16100 Kota Bharu, Kelantan, Malaysia

**Keywords:** energy saving, agreement technologies, building automation, negotiation, multi-agent systems

## Abstract

Nowadays, it is becoming increasingly common to deploy sensors in public buildings or homes with the aim of obtaining data from the environment and taking decisions that help to save energy. Many of the current state-of-the-art systems make decisions considering solely the environmental factors that cause the consumption of energy. These systems are successful at optimizing energy consumption; however, they do not adapt to the preferences of users and their comfort. Any system that is to be used by end-users should consider factors that affect their wellbeing. Thus, this article proposes an energy-saving system, which apart from considering the environmental conditions also adapts to the preferences of inhabitants. The architecture is based on a Multi-Agent System (MAS), its agents use Agreement Technologies (AT) to perform a negotiation process between the comfort preferences of the users and the degree of optimization that the system can achieve according to these preferences. A case study was conducted in an office building, showing that the proposed system achieved average energy savings of 17.15%.

## 1. Introduction

Our climate is undergoing a series of negative changes caused by human activity. Studies show that the concentration of greenhouse gases in the atmosphere is increasing, in addition to positive radiative forcing and global warming [1]. The combustion of fossil fuels releases large amounts of greenhouse gases. Large amounts of fossil fuels are still burnt in different industrial processes, in transport and in the production of electricity. The gas that is highly responsible for global warming is carbon dioxide, also known as CO${}_{2}$. Other gases that contribute to this process are methane, emitted by the action of microorganism at landfills and in agriculture (especially from the digestive systems of grazing animals), nitrous oxide from fertilizers, and gases used for cooling and industrial processes. Another factor that contributes to global warming is uncontrolled deforestation. Forests can absorb the carbon that is in the atmosphere; however, the clear-cutting of trees means that the percentage of CO${}_{2}$ that can be absorbed is lesser.

During the last few decades, society has become more aware of the need to use our energy resources efficiently. Government organizations are allocating more financial resources for the development of new energy policies and energy-saving solutions. The aim of these solutions is to slow down global warming and eventually restore its negative effects on the environment [2]. The European Union (EU) has identified energy efficiency as one of the pillars of sustainable growth strategies. It has defined three goals: (i) reduce greenhouse emissions by 20% (in comparison to 1990 consumption levels), (ii) 20% renewable energy in the EU, (iii) 20% improvement in energy efficiency. By 2050, the European Commission’s roadmap focuses on making the European economy more climate-friendly and energy efficient. This is why the next challenge is even more ambitious: by 2050, EU should have reduced its greenhouse gas emissions by 80% compared to 1990 levels [3]. However, first, it is necessary to reduce the emission of these gases by 40% in 2030 and 60% in 2040, [4,5].

To reduce the effects of global warming, it is necessary to make efficient use of energy resources and transform the electricity generation matrix into one that substitutes renewable and environmentally friendly sources for fossil fuels. Countries like Norway or Costa Rica exhibit an outstanding matrix of clean resources: water, geothermal, wind, solar and biomass energy, along with a minimum share of thermal generation, which functions as insurance coverage for contracted power generation [6]. Excluding geothermal energy, all of these renewable sources depend on climate.

However, the generation of clean energy is not the only important factor in making our energy use more efficient. It is also necessary to look at reducing the energy consumed by small consumers. There have already been several proposals in this regard. Many works have focused on obtaining information from sensors for the efficient management of HVAC (Heating, Ventilation, and Air Conditioning) and Lighting systems. In Spanish homes, the electricity used for lighting and other electric appliances is 32% of the total and 42% on space conditioning. These figures differ from other European countries where homes use 68% of total energy for space conditioning and 18% for lighting [7]. Thus, it is necessary to focus on these two areas if we want to obtain the highest energy savings possible. These two areas are also complementary, as many modern department stores can only be heated in winter with their lighting system and the heat produced by the users [8].

One of the works that most clearly addresses this problem is the one conducted by Nguyen and Aiello [9], the authors focused on classifying the factors that are relevant to users: desired temperature, desired lighting, occupation, occupation prediction, etc. Other authors Harle and Hopper [10] used occupancy information to control lighting and reduce temperature automatically when the building was not occupied. Padmanabh et al. [11] focused on office buildings and regulated both air and lighting when people were present in meeting rooms. Other works such as the one conducted by García et al. [12] optimized energy consumption by using gamification techniques together with the information collected through a Wireless Sensor Network (WSN) [12,13].

Former systems have succeeded at reducing the levels of energy consumption dramatically. They all achieved this by employing WSNs to collect environmental information. Although these systems considered the presence of people in the building, they did not adapt to user preferences, such as the desired temperature or degree of lighting. Thus, these systems may cause discomfort and users may feel discouraged from using them. It is therefore necessary to develop a system that considers all the household information in which the system is deployed as well as the comfort preferences of all its inhabitants. To this end, the system must behave like a social machine that allows all the participants to negotiate with each other and the system.

The goal of the research described in this article is to develop a new system for the analysis of parameters that influence the consumption of energy. On the basis of this analysis, the system will take decisions to optimize energy consumption in a dynamic environment by establishing a negotiation process with users. This work looks at the potential role of argumentation in next generation agreement technologies. Among the potential applications of argumentation in this area, the main focus of this work is on the specific domain of the redistribution of electricity consumption between lighting and temperature adjustment.

These are the main contributions of this paper: the developed system employs non-intrusive techniques in the collection of information, inhabitants are not in any way inconvenienced by the implemented system; negotiation between the inhabitants of a building; the system balances user preferences and energy optimization; lighting and temperature are adjusted automatically according to the negotiation agreement. All this made it possible to develop a system that combines information about each inhabitant’s comfort preferences and variables that influence energy consumption.

The rest of the article is structured as follows: Section 2 describes state-of-the-art proposals in the area of lighting and temperature control systems as well as studies on agreement technologies. Section 3 provides a full description of the system proposed in this work, including the functionality of each of its components. Section 4 details the case study and discusses the results obtained from an empirical implementation in a real scenario. Finally, Section 5 outlines the conclusions drawn from this research.

## 2. Related Work

To achieve our objective of designing an energy-saving system that negotiates environmental conditions with users, it is crucial that we understand how this can be done successfully. To this end, it is necessary to review the current state-of-the-art techniques, and such review will enable us to decide which techniques we will use to develop our system.

### 2.1. Traditional Home Energy Optimization Systems

Developments in the automation of homes and public buildings caused an increase in solutions that optimize the use of resources, such as water and electricity. Domotization allows to manage the consumption of devices connected to the power grid; it also makes it possible to control the way users interact with these devices, as well as other external factors that influence consumption. In buildings, HVAC and lighting systems account for the highest rates of electricity consumption. Since to a large extent, the consumption of energy in buildings depends on the activities of the people who live in them, it is necessary to detect and model occupation in buildings. Such knowledge will make it possible to save energy without renouncing the comfort of users.

Many researchers who work in the field of energy optimization have focused on the construction of WSNs. This is because WSNs facilitate the collection of information from which patterns in the behaviour of users can be identified and analysed [14,15]. These behavior patterns are necessary for performing simulations and executing decision-making algorithms; moreover, various data analysis techniques can be employed to predict occupancy [16]. Although previous researchers used WSNs to collect data that is related to factors that cause the consumption of energy; however, many factors were not taken into account [17,18,19].

Klein et al. developed a multi-agent comfort and energy simulation (MACES) system that simulates energy and comfort to model the management and control of building and occupant systems [20]. This system allowed for reducing energy consumption by 17% and achieved approximately 85% occupant satisfaction. This work is very similar to the proposal presented in this paper; however, it was only a simulation and was not implemented in a real case study. Yang et al. developed a multi-agent system to simulate management inside a building through the use of novel information technologies that optimized energy efficiency and comfort level. This system also has a personal agent, which learns about the behaviour of the environment by storing information on their interaction with the surrounding environment [21].

However, the energy-saving comfort control systems proposed so far do not optimise the energy consumed by the lighting system or the HVAC system because they do not control all the factors involved in the consumption of electricity. The systems that do control all the important consumption variables do not learn from user behaviour; this means that they cannot control the level of comfort and adapt to user preferences. Such systems are often disconnected by users as energy savings are achieved at the expense of their comfort. Thus, it is necessary to develop a complete system; one that optimises the factors that have the highest impact on the consumption of energy in a building and one that considers how consumption can be reduced while maintaining comfort and adapting to the preferences of inhabitants.

### 2.2. Agreement Technologies and Negotiation Systems

Agreement technologies (AT) refer to agents performing a negotiation process in which people and machines interact to reach an agreement that satisfies all the entities involved in the process [22]. The agreement must be consistent with the context in which it is made and it is established under a set of rules that must be fulfilled by the two entities. Each of these entities, by virtue of being autonomous, may choose to comply with the agreement or not, although the agreement assumes an obligation to reach the agreed objective. The entities participating in this paradigm must have certain characteristics, such as autonomy, interaction, mobility and transparency. Semantic adaptation, negotiation, argumentation, virtual organizations, real-time learning, and other possible technologies are the basis for defining, specifying, and verifying such systems [23]. This paradigm therefore makes it possible to consider the preferences of users while optimising electrical energy and reaching an agreement in a context (building or home).

This paradigm has been widely used in proposals where a consensus between users and machines is necessary to achieve a common goal. In the field of Cloud Computing, this paradigm has been used as a negotiation method for allocating virtual and physical resources between services to solve the problem of system overload [24]. This work provides the entities of the system with the capacity to participate in an argumentative dialogue in which the best solution is chosen before starting the negotiation. The entities make use of their experience and help each other avoid complex negotiation processes which, considering similar past experiences, are less likely to end in a successful allocation of resources. Villavicencio et al. used AT to create a movie recommendation system for groups of people [25]. The use of AT in this work made it possible to satisfy user preferences more equitably than traditional agreement methodologies. One of the novelties of this work is that the factors that threaten the validity of the agreement are considered. User groups were selected at random and any potential relationships between group members were ignored. However, in some cases, “similarities” between individual users (e.g., friendship, common tastes, etc.) within a group may affect the recommendations and therefore affect some groups’ satisfaction average [26].

### 2.3. Lighting and Temperature Automation

There are a wide variety of protocols for automating lighting, including Wi-Fi, ZigBee and Digital Addressing Lighting Interface DALI. The use of Wi-Fi entails a number disadvantages: (i) the scaling of large projects slows down the network bandwidth, (ii) security problems. The use of ZigBee implies high costs because drivers and controllers require a Zigbee wireless module. In addition, all the light bulbs need to have a Zigbee module, so the cost of their replacement is also higher. A bridge/hub for mobile devices is also needed so that they can act as controls. Zigbee has a slight compatibility problem even though it is an open standard [27]. Digital Addressing Lighting Interface (DALI) is an international standard (IEC 62386) and ensures full compatibility between devices from different manufacturers. It is a bidirectional regulation interface with a master–slave structure, where information flows from a controller (that operates as a master) to the lighting equipment (that operates solely as slaves), executing commands or responding to the received information requests. Communication by means of digital signals is carried out via a two-wire bus. These control wires (without polarity) can go together with the power supply cables (Phase, Neutral and Ground). This means that a five-wire hose (phase, neutral, ground and both DALI bus wires) is available for DALI installations [28].

Within the field of HVAC system automation, there are several commercial systems that can achieve average savings of up to 30%. There are a range of intelligent thermostat systems on the market, such as Tado${}^{\circ }$, Netatmo by Starck, Momit and Honeywell [29]. However, an intelligent heating system must understand the environmental context (building occupancy, user activities, building characteristics and climate) through an analysis of the data collected by sensors (ambient temperature, CO${}_{2}$ rate, humidity, state of occupancy, weather forecasts, etc.) and advanced algorithms (learning algorithms, prediction algorithms), whose main aim is to optimise energy consumption while maintaining occupant comfort. There are several open standard protocols for communication with the HVAC system including ModBus, LonWorks and BACnet [30]. These protocols have different sets of characteristics that make them suitable for different uses and applications [30]. According to a survey conducted on the Building Operating Management website in 2011 [14], 30% of respondents had at least one application that used Modbus, 40% used Lon-Works and 62% BACnet.

Building Automation and Control Network (BACnet) is the name used to refer to the ISO-16484-5:2007 standard. It is an open, non-proprietary communication protocol designed specifically for communication between heating, ventilation and air conditioning control systems; lighting control (DALI system), access control, and fire detection systems, along with their associated equipment. Building Automation and Control Network is the standard protocol in HVAC management; it is distinctive as it stands out for its scalability between costs, performance and system size, and also allows new innovations and features to be added at any time. Remote web access control also allows users to save time and money [31].

### 2.4. Multi-Agent Systems

Agents can be defined as autonomous software entities, capable of operating effectively in dynamic and open environments. Agents are often deployed in environments where they interact, and sometimes cooperate, with other agents (both individuals and software) who have potentially conflicting objectives [32]. A grouping of interacting agents that aims to achieve a common goal is called a multi-agent system (MAS). Multi-agent system can be designed to solve a very wide range of problems, and they operate according to a set of established rules and regulations. Groups of agents communicate and coordinate with each other to achieve a set objectives. Self-coordination refers to the way in which agents cooperate that the objective of minimising resource consumption is achieved. This is where communication plays an important role, it is an essential element of the MAS. These basic principles of communication and self-organization have been maintained from the time they were first defined [32]. The characteristics of this paradigm made its application beneficial in numerous fields such as real-time classification of human face images [33], WSN data fusion [34,35] or bioinformatics [36,37] but with a nexus that allows to work with, and analyze large amounts of data.

The ability of multi-agent systems to detect and react to changes in the environment, makes them ideal for obtaining data and performing adequate actions. Features such as extensibility and flexibility make it possible to add new functionalities or include other algorithms and sensors [38]. Due to their ability to communicate with more complex systems, they have been used in home automation. Numerous studies have made use of the properties offered by MAS to optimize energy consumption through the efficient management of the HVAC system exclusively [39,40,41] or even in conjunction with the management of lighting systems in buildings [21,42] and cities [43].

Agent based systems are an ideal methodology for building an argumentation model aimed at reaching an agreement between the system and the users. However, the system by itself will intend to reach an agreement that allows for optimizing energy use to the highest degree; although the decisions of this system would allow users to achieve considerable savings, the level of comfort in the building would be very low. In order to meet the users’ comfort preferences, it is necessary that the multi-agent system incorporate AT, so that an agreement may be reached between the actors present in the negotiation (the system and the users). In this regard, multi-agent systems offer a powerful model to represent the context (home or building) with an appropriate degree of complexity and dynamism.

The use of agent systems to simulate real world domains can provide us with answers to complex physical or social problems, which otherwise could not be understood. For example, the modelling of impact of climatic change on biological populations or the modelling of the impact of possible public policies on social or economic behaviour. In comparison to previous centralized approaches, multi-agent systems have provided us with faster and more effective methods of resource assignment in complex environments, such as management of public services networks.

The rest of the article describes the energy management system proposed in this article; it considers the advantages of previous proposals in this field and tries to solve the deficiencies of these systems in terms of their flexibility, automation and pattern learning.

## 3. System Overview

This section describes the proposed system, with a special focus on the negotiation part on the conditions to which the environment must conform and which is carried out between the agents representing the users of the environment, since the part of the system that allows the system to perform the actions that allow optimization as well as communication with the HVAC and lighting system, is based on a previously published work [44].

Each user in the environment (the environment is defined as every room in the building in which the system has been deployed) has an agent that retains their preferences. These preferences can be established on a mobile application specially developed for this system. The preferences include the following information: (i) lighting preferences in the working environment that are defined by a percentage value between 0 and 100, together with a threshold established somewhere between 5% and 10%; (ii) heating preferences in the working environment, defined by a value in degrees Celsius (${}^{\circ }$C) together with a threshold of between 2 and 5 ${}^{\circ }$C; (iii) a schedule, with the possibility of determining preferences (i) and (ii) for each time zone; and (iv) three types of user profiles in negotiations, which may be safe if the user wants the environmental conditions to suit their preferences, economic if the user prefers to consume as little as possible or neutral if the user wants a balance between their preferences and energy-saving measures, this enables the system to diverge a little from these preferences. The use of these three profiles allows the system to avoid a negotiation situation that does not allow the correct establishment of a comfort preference agreement.

In addition, when a user enters an environment, their Comfort Value (CV) is automatically calculated, based on their preferences and the current environmental conditions. The CV ranges between 0% and 100%, where 100% are the conditions of the environment as defined by the user’s preferences. If the user’s profile is economic, the brightness or temperature values are not included in user preferences and their CV ≥80% percent lower than the CV. Users with economic profiles will not participate in the negotiation. The CV of users with neutral profiles is also CV ≥80%. Otherwise, the User Agent that has just entered the system initiates a negotiation with the User Agents that are already active in the system.

The next subsections describe the negotiation process. The elements that make up the MAS architecture are identified.

### 3.1. Obtaining Context Data

This section details the way in which the system collects the lighting data and temperature values.

**Figure 1:**
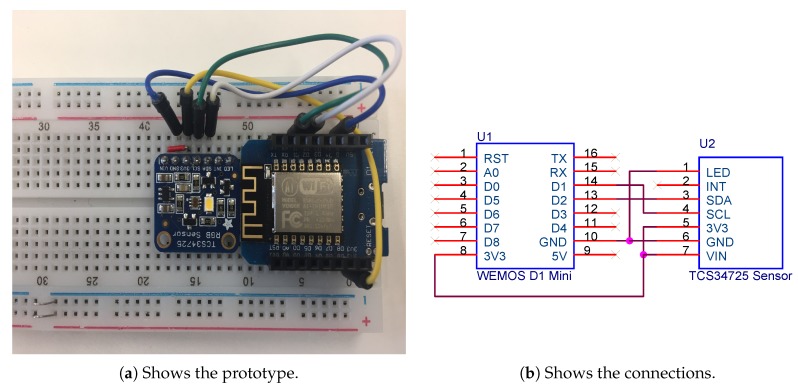
Prototype of the light sensor system.

**Figure 2:**
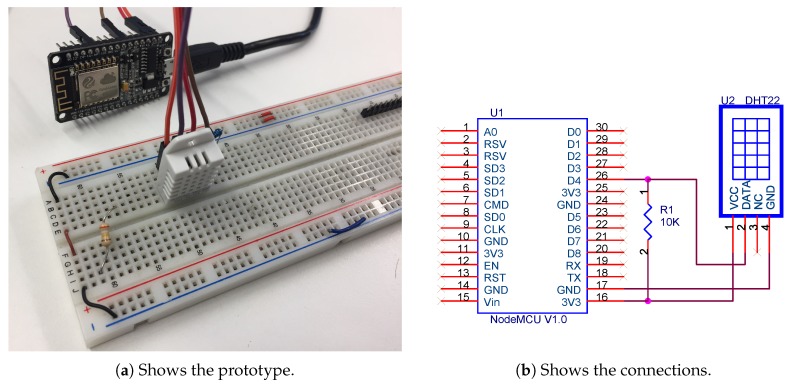
Prototype of the temperature acquisition system.

- *Color light-to-digital converter with IR (Infrared Radiation) filter connected to a We-Mos D1 mini module (WEMOS, China) (ESP8266-based)*: This sensor allows for measuring the amount of lux in an office (1 lx = 1 lm/m${}^{2}$), and it determines if the light is clear and the color in RGB format is obtained (Figure 1). This sensor has the IR blocking filter that minimizes the IR spectral component of the incoming light, providing much more accurate color measurements than those read by most sensors. The sensor also has an incredible 3,800,000:1 dynamic range with adjustable integration time and gain so it is suited for use behind darkened glass. This sensor works when connected to any microcontroller with I${}^{2}$C and it can obtain four channel readings (red, green, blue and clear).
- *Temperature sensors*: Temperature is an important factor that is included in the agreement algorithm; therefore, it is necessary for the system to obtain the temperature values inside the rooms. This is done thanks to the DHT22 sensor that is used to obtain temperature and humidity values, this sensor can be seen in Figure 2. In cases where the walls of the offices face the outside, the system must also collect outdoor temperature. The importance of obtaining outdoor temperature data lies in the fact that the temperature is one of the key factors in the optimization of energy in households. This sensor allows us to measure temperatures between −40 ${}^{\circ }$C and 125 ${}^{\circ }$C with an accuracy of 0.5 ${}^{\circ }$C, humidity measurement between 0% and 100% with an accuracy of 2–5% and a sampling frequency of 0.5 Hz.

**Figure 3:**
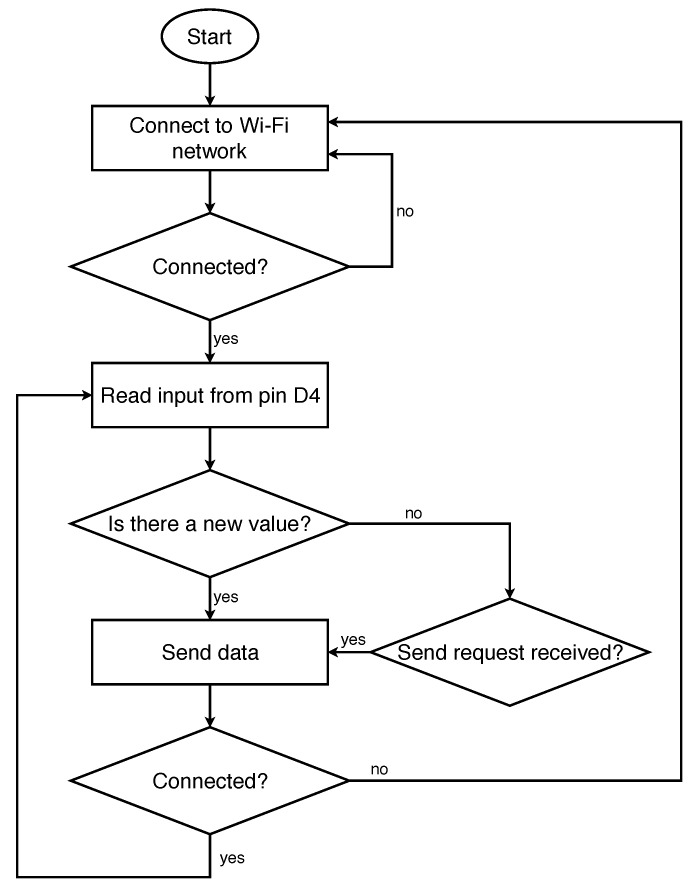
Software running in the temperature acquisition device.

The device’s software has been developed in Arduino and its operation is described with the flowchart presented in Figure 3. Moreover, information is read synchronously, so that the status “Read input from pin D4” stops the execution of the software until new data is available in the input.

### 3.2. Multi-Agent System Schema

The MAS that supports the system was developed using JADE [45]. The developed agents implement five different types of roles:

- Environment Agent: the role of these agents is to provide information on the status of a single environment. A new Environment Agent is created for every environment that is introduced into the system.
- Sensor Agent(s): the amount of sensor agents created in the system depends on the characteristics of the environments registered in the system. These agents are responsible for providing information on the state of the brightness and temperature of the environment. Moreover, they detect when a user enters or leaves the environment (through the connection of their mobile device with the access point). There must be at least three Sensor Agents in every environment: one for measuring brightness, one for temperature and one for the identification of users in the environment.
- Preference Agent: inhabitants use a mobile application to establish their preferences, the preference agent is in charge of keeping track of these preferences. The application is described further on in this paper. The users’ lighting and temperature preferences are specified, along with a comfort range.
- User Agent(s): these agents are responsible for the negotiation process described below. Each user agent is associated with a single user in the environment.
- Negotiation manager Agent: a Negotiation manager Agent is created in each environment in order to manage the negotiation process between User Agents.

**Figure 4:**
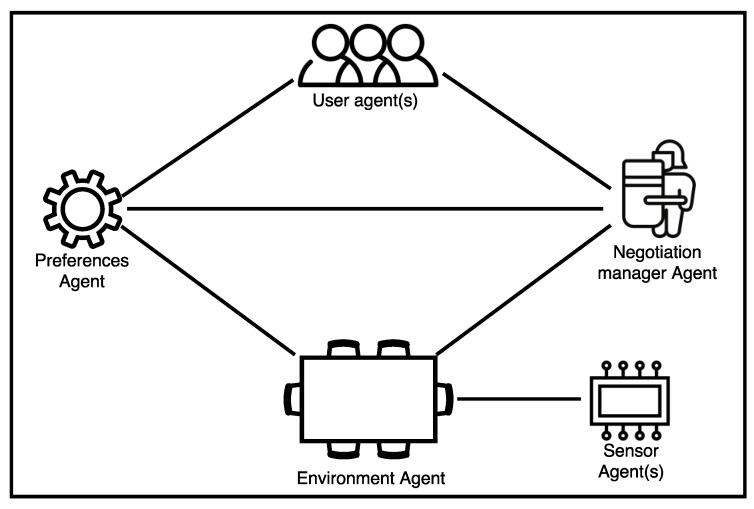
Multi-Agent System (MAS) schema.

An overview of the interaction between the agents described above is shown in the Figure 4.

#### 3.2.1. Negotiator Agent Model

**Figure 5:**
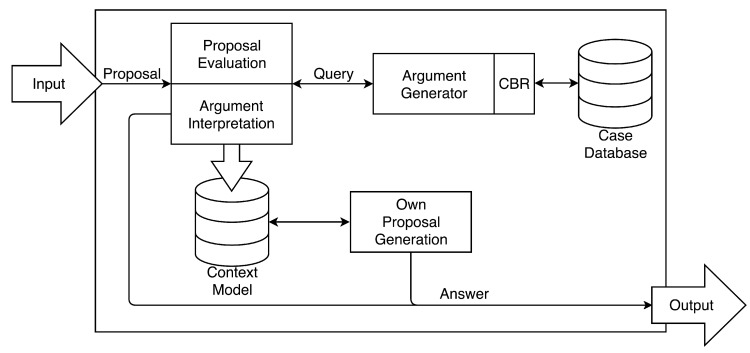
Argumentation-Based Negotiator (ABN) structure.

This section presents the structure of the negotiator agents. Figure 5 represents the structure of the User agent, an Argumentation-Based Negotiator (ABN), which is a fundamental trait. As shown, the agents have the possibility of explicitly exchanging meta-information.

The most important elements of the proposed ABN structure are presented below.

#### 3.2.2. Negotiation Description

One of the most important parts of the system is the negotiation between each User agent participating in the same system environment. The objective of the negotiation is to determine, from the preferences of each party, the conditions that are to be established in that environment.

Negotiation can begin when one of the following three events occurs: (i) a User agent with established preferences changes its status to active according to the defined schedule; (ii) a User agent changes its preferences in real time or changes its time zone as defined in the preferences; (iii) a User agent leaves the environment, changing its status to inactive. In addition to events (i) and (ii), it is also necessary that the conditions in the environment be different than those desired by users. In the case of event (iii), the negotiation always takes place between the User agents with an active status in the environment.

When the negotiation begins, each of the agents that are involved takes a position that suits its needs.

#### 3.2.3. Negotiation Mechanism

When defining an agreement-based negotiation model in which arguments are used, it is first necessary to determine the number of mechanisms that support the negotiation process itself. The most important mechanisms are communication language and domain language.

To begin, the FIPA ACL (Foundation for Intelligent Physical Agents’ Agent Communication Language) [46] is selected as the primary communication language because of its semantic capabilities, as it includes locutions to express acceptance, rejection, proposal applications, requests, inquiries, statements, declarations, etc. Communication was established through the PANGEA platform [47,48], which allows for cross-platform distributed development and disengages the specific functionality of the application of basic functions, such as access to data or norms of communication between agents. For this negotiation, four types of locution on FIPA-ACL are to be used: (i) inform: desire_to_change (L3), desire_not_to_change (L4), prefer_to_change (L5), prefer_not_to_change (L6), withdraw_dialogue (L11); (ii) propose: open_dialogue (L1); agree_to_change (L9); (iii) accept-proposal: enter_dialogue (L2), agree_not_to_change (L10); (iv) refuse: refuse_to_change (L7), refuse_not_to_change (L8).

Once the communication language is defined, it is necessary to define a domain language, allowing the passage of meta-information separately or together with other locutions. To this end, we must define an ontology compatible with FIPA in order to carry out the decision-making process that will determine the conditions of the environment. Its class structure is defined in the Table 1.

The structure is composed of two abstract classes (Concept and Predicate). The other classes are defined as shown in the diagram. For a better understanding, the type attributes, constraint and valuation must be defined.

To begin, (i) attributes reflect parameters that are associated with the environment: the current light level, the current temperature and all the preferences (change requirements) received by the active User Agents. The value reflected by (ii) constraints refers to the User Agent preferences, including the light percentage and the range, the temperature value and range, the type (economic, safe or neutral) and the CV, which represents the user comfort with the current environment conditions. Finally, (iii) valuation provides the User Agent with a CV if the preferences of the other active User Agents in the environment are taken into account.

#### 3.2.4. Use of Dialogue Model Locutions

To facilitate the understanding of the dialogue model locutions, an example is shown below. Sender and receiver fields must be included in ACL messages, but they are not included to facilitate the reading. The example shows a real case where two User Agents are selected from the same environment (environmentID = “241”) to show a real example of the values.

The environment has the following attributes: brightness level 83%, temperature 21 ${}^{\circ }$C and there are five other active User Agents with the following preferences: User Agent 1: 80% ± 15%, 22 ${}^{\circ }$C ± 2 ${}^{\circ }$C; User Agent 2: 60% ± 20%, 23 ${}^{\circ }$C ± 3${}^{\circ }$C; User Agent 3: 95% ± 5%, 20 ${}^{\circ }$C ± 1 ${}^{\circ }$C; User Agent 4: 82% ± 5%, 20 ${}^{\circ }$C ± 1 ${}^{\circ }$C; and User Agent 5: 75% ± 10%, 20.5 ${}^{\circ }$C ± 2 ${}^{\circ }$C.

On the other hand, the new User Agent (6) has the following constraints: 90% ± 10%, 26 ${}^{\circ }$C ± 3 ${}^{\circ }$C and its profile type is safe, so when it becomes active, it enters into negotiation with each active agent in the environment. In addition, in the evaluation of the preferences of each of the active User Agents in the environment is the following: User Agent 1: 87%, User Agent 2: 79%, User Agent 3: 85%, User Agent 4: 84%, User Agent 5: 81%. The comfort value algorithm was applied in this evaluation, calculating the weight of temperature as four times greater than the brightness. This shows that temperature has a greater influence on the comfort of inhabitants.

**Figure 6:**
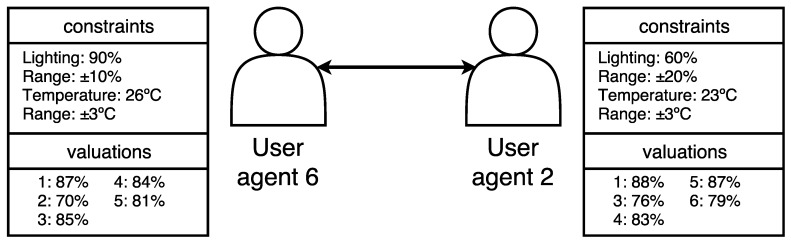
Sample of constraints and valuations table for two agents.

To present the example with each of the possible expressions, the constraints of another User Agent (User Agent 2), also of the safe type, are described: 60% ± 20%, 23${}^{\circ }$C ± 3${}^{\circ }$C, with valuations: User Agent 1: 88%, User Agent 3: 76%, User Agent 4: 83%, User Agent 5: 87%, User Agent 6: 79% (see Figure 6).

- L1: open_dialogue(.): propose (AgentAction (Open_dialogue: environment “241”)).
- L2: enter_dialogue(.): accept-proposal (AgentAction (Open_dialogue: area “241”)).
- L3: desire_to_change(.): inform (Desire_to_change (Environment: attributes “83, 21; 80, 15, 22, 2; 60, 20, 23, 3; 95, 5, 20, 1; 82, 5, 20, 1; 75, 10, 20, 2”: constraints “90, 10, 26, 3”)).
- L4: desire_not_to_change(.): inform (Desire_not_to_change (Environment: attributes “83, 21; 80, 15, 22, 2; 60, 20, 23, 3; 95, 5, 20, 1; 82, 5, 20, 1; 75, 10, 20, 2”: constraints “60, 20, 23, 3”)).
- L5: prefer_to_change(.): inform (Prefer_to_change (Environment: attributes “83, 21; 80, 15, 22, 2; 60, 20, 23, 3; 95, 5, 20, 1; 82, 5, 20, 1; 75, 10, 20, 2”: constraints “90, 10, 26, 3”: valuation “(0.87, 0.79, 0.85, 0.84, 0.81)”)).
- L6: prefer_not_to_change(.): inform (Prefer_not_to_change (Environment: attributes ““83, 21; 80, 15, 22, 2; 60, 20, 23, 3; 95, 5, 20, 1; 82, 5, 20, 1; 75, 10, 20, 2”: constraints “60, 20, 23, 3”: valuation “0.88, 0.76, 0.83, 0.87, 0.79”)).
- L7: refuse_to_change(.): refuse (AgentAction (Change (Environment: attributes “83, 21; 80, 15, 22, 2; 60, 20, 23, 3; 95, 5, 20, 1; 82, 5, 20, 1; 75, 10, 20, 2”))).
- L8: refuse_not_to_change(.): refuse (AgentAction (Not_change (Environment: attributes “83, 21; 80, 15, 22, 2; 60, 20, 23, 3; 95, 5, 20, 1; 82, 5, 20, 1; 75, 10, 20, 2”))).
- L9: agree_to_change(.): propose (AgentAction (Agree_to_change (Environment: attributes “83, 21; 80, 15, 22, 2; 60, 20, 23, 3; 95, 5, 20, 1; 82, 5, 20, 1; 75, 10, 20, 2”))).
- L10: agree_not_to_change(.): accept-propose (AgentAction (Agree_not_to_change (Environment: attributes “83, 21; 80, 15, 22, 2; 60, 20, 23, 3; 95, 5, 20, 1; 82, 5, 20, 1; 75, 10, 20, 2”))).
- L11: withdraw_dialogue(.): inform (Withdraw_dialogue: environment “241”).

Given the data in this example and the characteristics of the two User Agents described above, if they had to negotiate, an agreement would be reached quickly. The reason for this is that User Agent 2 has obtained the lowest rating due to its very different lighting preferences. User Agent 6 and User Agent 2 are the most similar in terms of temperature, and even overlap in their range. In addition, their lighting preferences overlap by 80%. Therefore, the agreement reached by these two agents would be 80% in terms of lighting conditions and a temperature of 24.5 ${}^{\circ }$C. Despite this, the agreement must be reached between all active user agents that are participating in the negotiation, in the above case; therefore, the negotiation would continue with the rest of the agents.

In the event that the rest of the User Agents are neutral and the negotiation ends when the securities are within their range, the negotiation will end and the conditions of the agreement will be the conditions to which the environment will be subject. On the other hand, it can be observed that the values they have established are not within the preferences of the rest of the User Agents (1, 3, 4 and 5).

If any of the other agents had an economic role, it would have preference in the negotiation with User Agent 6, since in its preferences it aims to increase the temperature of the environment, assuming a higher energy consumption. In this way, their arguments could be imposed by the Negotiation Manager Agent if no agreement is reached. The Negotiation Manager Agent coordinates the negotiations between each pair of User Agents and determines what conditions are to be established based on the agreements reached between them.

**Figure 7:**
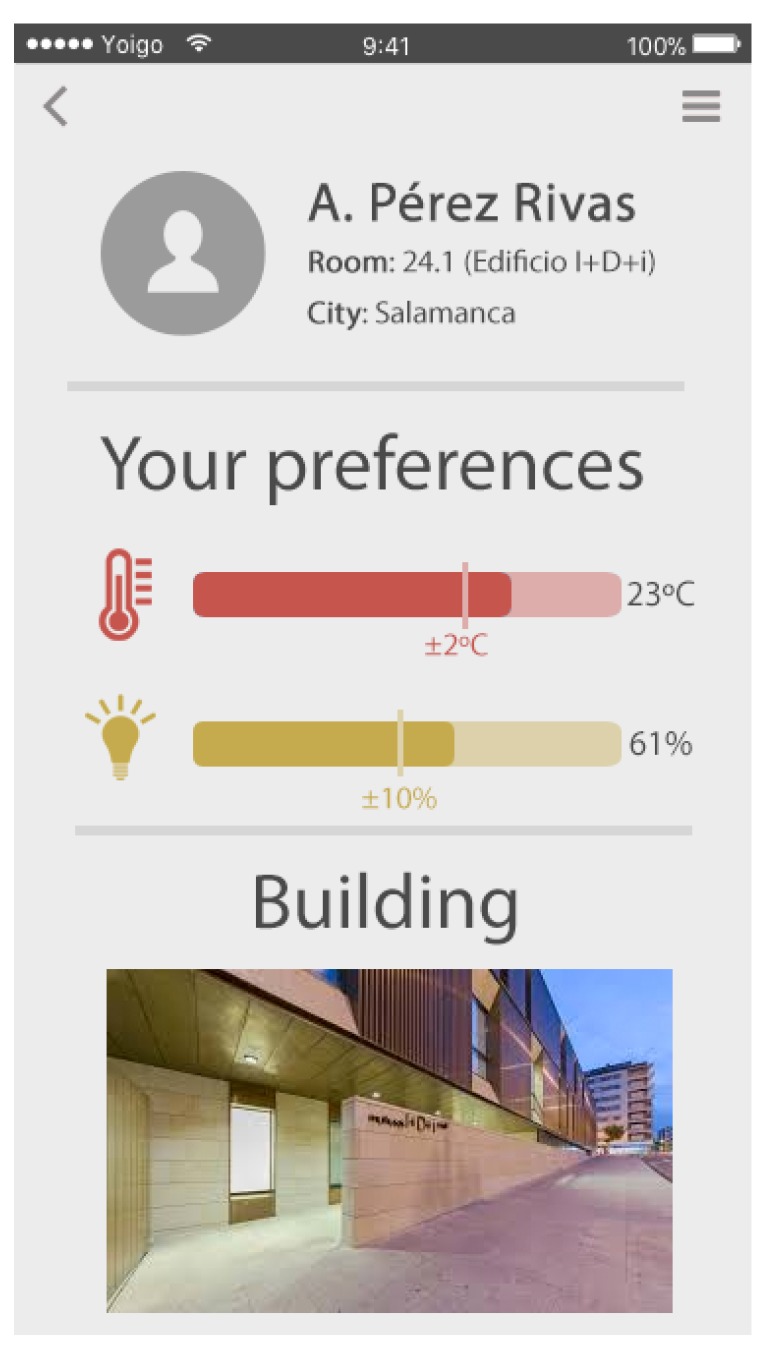
Mobile application for establishing user preferences.

Users can set their preferences through a mobile application shown in the Figure 7. Using each user’s mobile device allows the system to detect when they access the environment automatically through the MAC (Media Access Control) address of their devices, and the OpenWrt-based access point executes a Python script that detects changes in the connected devices and alerts the Negotiation Manager Agent to evaluate whether the negotiation should start.

## 4. Case Study

It is necessary to validate if the system is capable of performing a negotiation process that establishes the comfort preferences (temperature and lighting) and at the same time reduces energy consumption. To perform a valid evaluation of the system and to demonstrate that the system allows for energy savings without affecting user comfort, different control groups in the case study setting are chosen for validation.

The International Performance Measurement and Verification Protocol (IPMVP), which is the basis of the common European ICT PSP Methodology for calculating energy savings in buildings, proposes four different procedures for settling in the non-intervention consumption [49,50]. The first method consists of isolating the key parameters, the second method is the isolation of all parameters, the third method is whole facility and finally the fourth method consists of calibrated simulation. Out of these four options, only the third option is useful for measuring the utility of the proposed system, since it does not have to assume a constant energy demand; also, the variation in demand can be modelled with precision. The configuration performed in the case study is detailed below.

### 4.1. Experimental Set-Up

In order to verify the efficiency of the multi-agent system that allows agreements between users in terms of comfort under a process of energy optimisation, an experiment was designed consisting of two groups (Control Group and experimental group) and two phases (Baseline: energy consumption monitoring phase and Intervention Period: benefit contrast phase, phase that certifies the existence or not of a reduction in energy consumption).

**Figure 8:**
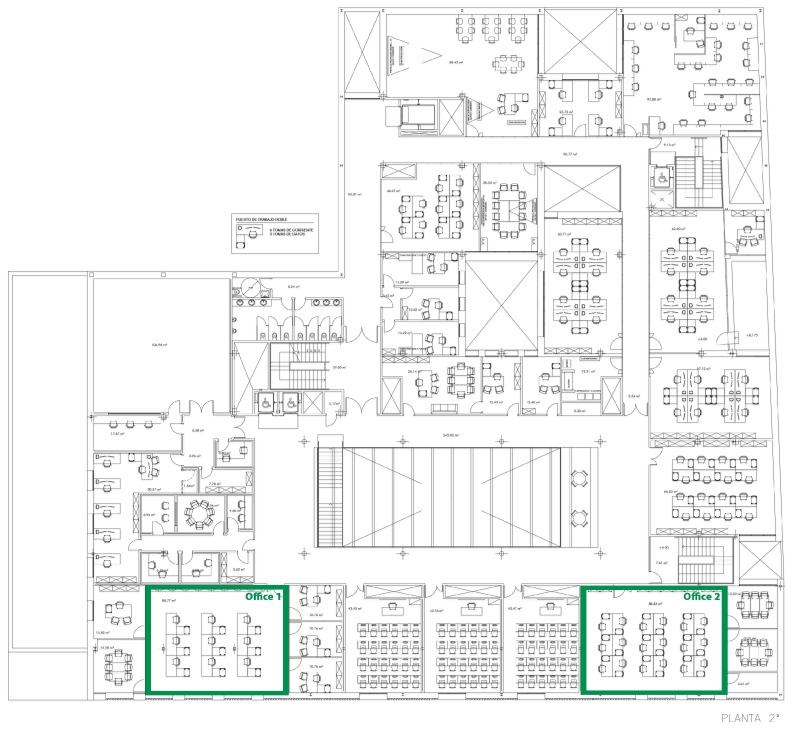
Plan of the building in which the two offices in which the case study was carried out are located. Office 1 is the control group and Office 2 is the experimental group.

The experiment was conducted in the R&D&I Building of the University of Salamanca, which is shown in Figure 8. The two offices in which the case study was conducted share the same architectural design characteristics, as shown in Table 2. By conducting the case study in two offices with the same technical characteristics, the proposed system can be validated independently of the variables that affect energy consumption: same outdoor temperature, same number of hours of solar radiation, same number of holidays or same number of working hours of employees, as well as other factors.

The experiment lasted two months and took place from October 2 to December 4. The choice of this period is based on the fact that in this period the maximum and minimum temperatures do not vary significantly from one week to the next and the number of holidays per month is similar.

As detailed above in the Baseline period (October 2–November 2), consumption was monitored only once in both offices. In the Intervention period (November 2–December 4), both groups were monitored and the system was employed in Office 2. Office 1 is the control office that allows for measuring whether consumption grew, decreased or remained from the first month to the second month. This will be the reference point to know if the experimental group shows a reduction in energy consumption.

**Figure 9:**
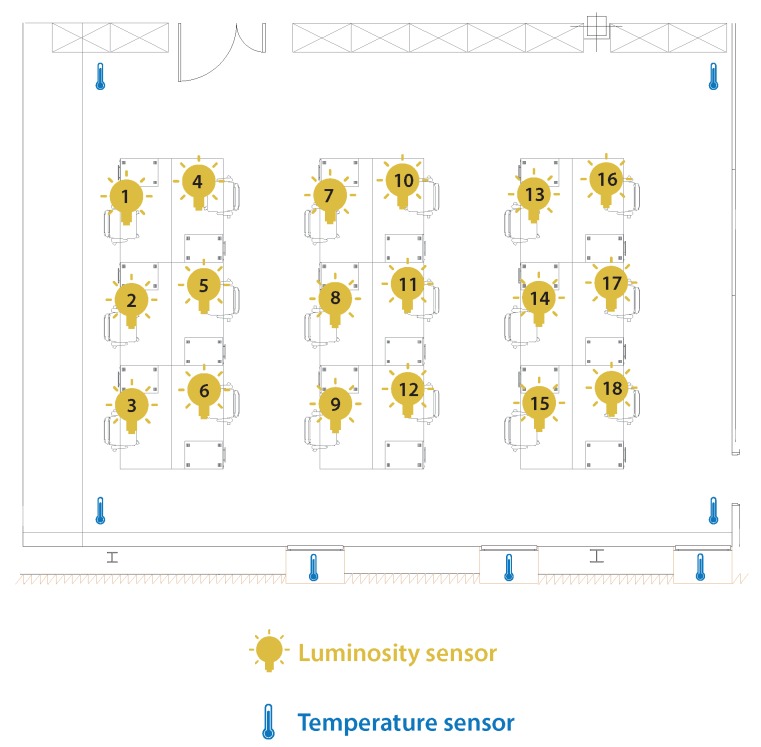
Example of the display of sensors in Office 2.

Figure 9 shows how the sensors used for data collection are deployed in one of the offices. A presence sensor was installed at each workstation, an outdoor temperature sensor on each window and an indoor temperature sensor in each corner of the office.

### 4.2. Empirical Results

This section outlines the results rendered by the system in the conducted case study. One of the features of the developed system is that it adapts itself to the conditions and characteristics of the context (location and users), the two case study offices in which the conditions in temperature and lighting were variable over time. During the month-long Baseline period the system collected data from both offices. In the Baseline period, the system collected the average temperature and lighting values as well as energy consumption. This provided us with knowledge on the values of the variables that play a role in the negotiation and energy optimization process. During this period, the system has only monitored the value of these variables in both offices. In the intervention period, the actors in charge of the negotiation process have played an important role in establishing comfort preferences and obtaining energy savings.

In Table 3, it can be seen that, in the Baseline period, both Office 1 and Office 2 have a similar consumption (43.69 Wh and 44.00 Wh). This has been caused by almost identical indoor brightness, and outdoor and indoor temperature conditions. On the other hand, the Intervention period shows that the outdoor and indoor temperature and lighting conditions remain almost the same. However, it is also noted that in Office 2, the system assumed a significant decrease in electricity consumption (it went from 40.19 Wh in Office 1, to 36.45 Wh in Office 2). It is also noted that the indoor temperature of Office 2 is higher than the indoor temperature of Office 1, but the indoor lighting is lower. We induce that this is due to user preferences and that the lower level of lighting has caused consumption to reduce.

In Table 4, we can read the energy consumed in each stage (Baseline and Intervention period) of the case study. It can be seen that the consumption in the experimental group has been reduced by 17.44% and the control group by 5.39%, which means that from the Baseline period to the Intervention Period, there has been a reduction in energy consumption. This is because the last week of December is a holiday period (Christmas), but we need to verify that the large percentage of reduction that has occurred in the control group is due to the use of the system.

### 4.3. Statistical Validation

To verify that it is possible to obtain a reduction in energy consumption, Student’s *t*-test and Levene’s test have been applied to the results. They assessed the difference of means (electrical consumption in kWh) and variances between the baseline and intervention period. In the tests, the level of α was established as 0.05 and the size of the samples was n1 = 30, n2 = 30, which was equivalent to the number of days of each period, where F is Levene’s Test for equality of variances and *t* is the *t*-test for equality of means. The established H0 hypothesis established that the average energy consumed in each office before the deployment of the proposed system is equal to the average energy consumed in each office with the system performing negotiation processes; H1 stated that the average energy consumption in the office is lower when the negotiation process takes place between the comfort preferences of the users and the economic position of the proposed system. If the *p*-value is less than 0.05 (significance value), the H0 hypothesis is rejected and H1 is true, so the system consumes less electricity when using the system with agreement technologies.

**Figure 10:**
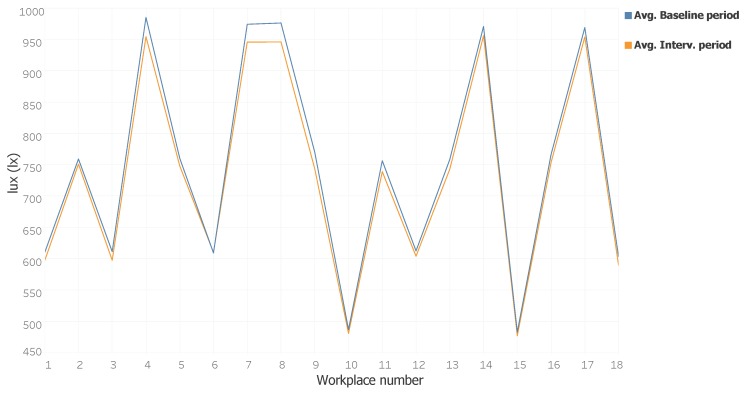
Example of the display of sensors in Office 2.

In Table 5, we can see the average light intensity values for each workstation during the case study. We can see a slight decrease in the values collected during the Intervention Period. This is because, in the Baseline period, users established their lighting values above their minimum light intensity preferences. Then, when negotiations have taken place with the system, the system was able to decrease the light intensity per station a little, which has a knock-on effect on the consumption of electricity. Figure 10 shows a more visual comparison of the average values from the two periods. It can be seen how the reduction of light intensity per workstation was slightly lower at all workstations and almost unnoticeable.

In Table 6, we can observe the results of both tests and how the energy consumption is significantly lower in the intervention period, with a *p*-value of 0.000. These results demonstrate the contribution of this technologies in obtaining energy savings.

## 5. Conclusions

This work takes a novel approach to reducing energy consumption by negotiating office users’ comfort preferences with the decisions of the system. Agreement Technologies and a Wireless Sensor Network have both been implemented in the agents within the developed multi-agent system. The WSN collects environmental data and the use of AT makes it possible to meet users’ comfort needs. These comfort agreements can be made thanks to AT, which performs a negotiation process between the user preferences and the decisions made by the system. Through the deployment of agents, the MAS is capable of monitoring, negotiating and arguing using the preferences coming from users and the optimization activities that the system identifies. Once the preferences have been established among users, they are negotiated with the system, which has an economic position whose objective is to satisfy the comfort of the users at the same time as the energy consumption decreases.

As a result of the implementation of the system, consumption was reduced by 17.44% in comparison to usual consumption. These results demonstrate that it is possible to reduce the consumption of energy while satisfying the comfort preferences of a group of office workers. To verify that savings resulted from the application of the system and not from external factors, Student’s *t*-test and Levene’s test have been applied with satisfactory results. There was also a reduction of 5.39% in the control group, however, by applying the previously mentioned tests to the results, it was possible to prove that the reduction was due to the lack of activity during the Christmas holiday period. Furthermore, this study demonstrates that users do not perceive when the temperature is slightly lower than their preferences, this allows the system to reduce the energy consumption of the HVAC system. Another interesting fact is that, in the negotiation process, users preferred to give up a few degrees of temperature but not lose light intensity.

## Figures and Tables

**Table 1:** Negotiation ontology.

**Concept**
AgentAction - Open_dialogue: environmentID (Integer) - Agree_to_change: proposal (Environment instance) - Change: proposal (Environment instance) - Not_change: proposal (Environment instance)
Environment: attributes (String)
Change Requirement: constraints (String)
Change Requirement Valuation: constraints (String): valuation (String)
**Predicate**
Desire_to_change: environment (Environment instance): change requirement (Change Requirement instance)
Desire_not_to_change: environment (Environment instance): change requirement (Change Requirement instance)
Prefer_to_change: environment (Environment instance): change requirement valuation (Change Requirement Valuation instance)
Prefer_not_to_change: environment (Environment instance): change requirement valuation (Change Requirement Valuation instance)
Withdraw_dialogue: area (String)

**Table 2:** Technical characteristics of the offices that are part of the case study.

	Office 1	Office 2
**Cardinal situation**	SE	SE
**Office dimensions**	88.83 m${}^{2}$	88.83 m${}^{2}$
**Number of workplaces**	18	25
**Number of windows**	1	1
**m2 of window surface**	6.3 m${}^{2}$	6.3 m${}^{2}$

**Table 3:** Daily average values of the factors in each phase of the case study.

		Office 1 (Control Group)	Office 2 (Experimental Group)
Baseline period	Avg. Outdoor Temp (${}^{\circ }$C)	2.52	3.54
	Avg. Indoor Temp (${}^{\circ }$C)	22.29	22.75
	Avg. Luminous flux (lx)	689	673
	Energy Consumption (Wh)	43.69	44.00
Intervention period	Avg. Outdoor Temp (${}^{\circ }$C)	7.48	7.48
	Avg. Indoor Temp (${}^{\circ }$C)	19.98	21.64
	Avg. Luminous flux (lx)	747	575
	Energy Consumption (Wh)	40.19	36.45

**Table 4:** Total consumption in Wh during the month of the Baseline period, during the month of the Intervention period and the difference in the consumption and savings between the two periods.

	Office 1 (Control Group)	Office 2 (Experimental Group)
Baseline period–Consumption (Wh)	43.6929	44.0033
Intervention period—Consumption (Wh)	40.1943	36.4548
Difference (Wh)	3.4986	7.5485
Savings (%)	8.00	17.15

**Table 5:** This table shows the average number of lumens per workstation per week for Baseline Period and for Intervention Period in Office 2.

		Workplace																		
		1	2	3	4	5	6	7	8	9	10	11	12	13	14	15	16	17	18	Avg.
**Baseline Period**	**Week 1**	596	747	596	959	747	596	956	959	747	475	747	596	742	959	475	747	959	596	733.28
	**Week 2**	632	771	621	1001	779	620	1002	999	809	500	768	631	771	990	493	791	982	612	765,11
	**Week 3**	612	765	615	978	763	612	972	978	766	491	756	624	765	974	482	777	970	610	750.56
	**Week 4**	598	753	612	1002	752	608	966	968	757	481	754	598	755	959	481	749	964	594	741.72
**Interv. Period**	**Week 1**	588	732	575	948	741	596	934	942	725	481	736	578	739	963	480	736	847	577	723.22
	**Week 2**	625	761	604	950	765	611	957	957	760	502	754	624	745	948	484	744	942	615	741.56
	**Week 3**	596	765	602	960	736	636	955	948	745	477	732	611	745	968	471	780	970	601	738.78
	**Week 4**	578	745	608	959	750	596	936	936	743	462	733	602	742	944	472	753	956	564	726.61

**Table 6:** Results of the Student’s *t*-test and Levene’s test performed in the office of case study. Difference of means (electrical consumption in kWh) and variances between the data obtained in baseline and intervention periods.

	Baseline Period		Intervention Period					
	Mean (kWh)	Stdr. Deviation (kWh)	Mean (kWh)	Stdr. Deviation (kWh)	*t*	Sig. (2-tailed)	F	Sig.
Office 1 (Control Group)	43.6929	1.15606	40.1943	3.68999	4.146	0.000	38.635	0.000
Office 2 (Experimental Group)	44.0033	0.58033	36.4548	1.65132	19.763	0.000	20.027	0.000
